# Mental health disorder in chronic liver disease: a questionnaire survey

**DOI:** 10.3389/fpsyt.2024.1469372

**Published:** 2024-10-25

**Authors:** Jiang Long, Xiong Pei, Wei Jiang, Xiaoling Wang, Dongbo Wu, Xiangdong Tang, Taoyou Zhou

**Affiliations:** ^1^ Mental Health Center, West China Hospital, Sichuan University, Chengdu, China; ^2^ Center of Infectious Diseases, West China Hospital, Sichuan University, Chengdu, China; ^3^ Division of Infectious Diseases, State Key Laboratory of Biotherapy and Center of Infectious Diseases, West China Hospital, Sichuan University, Chengdu, China; ^4^ Center of Infectious Diseases, West China Hospital, Sichuan University/West China School of Nursing, Sichuan University, Chengdu, China; ^5^ Outpatient Department, West China Hospital, Sichuan University, Chengdu, China; ^6^ Sleep Medicine Center, Mental Health Center, and State Key Laboratory of Biotherapy, West China Hospital, Sichuan University, Chengdu, China

**Keywords:** chronic liver disease, mental health, epidemiology, risk factor, affective disorder

## Abstract

**Background:**

The mental health of patients with chronic liver disease (CLD) warrants greater attention and understanding, especially concerning its risk factors.

**Method:**

Patients from our hospital’s hepatology clinic were consecutively enrolled and completed a questionnaire assessing anxiety, depression, and sleep quality using the GAD-7, PHQ-9, and PSQI scales, respectively. Reliability and validity were evaluated with Cronbach’s α and Kaiser-Meyer-Olkin (KMO). Continuous and categorical variables were analyzed using the Mann-Whitney U and Chi-square tests. Univariate and multivariate logistic regression were used to identify risk factors, while restricted cubic spline (RCS) were used to explored non-linear associations. Subgroup analyses were stratified by sex, age, and education.

**Result:**

A total of 1030 questionnaires were collected, and after quality control, 1003 were included. 56.2% (564/1003), 53.2% (534/1003), and 67.4% (676/1003) individuals had anxiety, depression, and sleep disorders. Differences in age, gender, and education level were observed (*P*<0.05). Subgroup analysis showed similar demographic trends. Univariate and multivariate regression analysis found age negatively correlated with anxiety (OR=0.98, 95%CI=0.97-0.99, *P*=0.02) and depression (OR=0.98, 95%CI=0.96-0.99, *P*<0.001), but positively correlated with sleep disorders (OR=1.03, 95%CI= 1.01-1.05, *P*< 0.001); males are less prone to anxiety (OR=0.68, 95%CI=0.52-0.88, *P*=0.004) and sleep disorders (OR=0.72, 95%CI: 0.55-0.94, *P*=0.02); university degree is more susceptible to depression (OR=1.36, 95%CI=1.04-1.77, *P*=0.02) and anxiety (OR=1.45, 95%CI=1.11-1.89, *P*=0.007). RCS analysis suggested a linear relationship between the age and affective disorders among different population.

**Conclusion:**

Young individuals, female, and those with higher education are more vulnerable to mental health, warranting increased attention.

## Introduction

Mental health, including anxiety, depression, and sleep disorders, encompass a wide range of conditions that affect mood, thinking, and behavior (1, 2). These conditions can occur as separate issues or may overlap, significantly impacting daily lives and well-being (3, 4). Recently, epidemiological studies showed that mental health disorders are on the rise globally. A study by Liu, Sitong et al. revealed that the anxiety and depression in Chinese residents was 19.7% and 45.02% respectively (5). During the COVID-19 pandemic, the rates of mental health symptoms among Chinese were 27.9% (95% CI, 27.5%-28.2%) for depression, 31.6% (95% CI, 31.2%-32.0%) for anxiety, and 29.2% (95% CI, 28.8%-29.6%) for insomnia (6). The World Health Organization (WHO) has highlighted mental health as a critical global issue, with millions of people affected each year, and the numbers are expected to increase (7, 8). The harm of mental health disorders extends beyond individual suffering but also impose economic burdens on healthcare systems and societies.

Chronic liver disease (CLD) is a major global health threat and has become a leading cause of human mortality. Despite advances in treatment and prevention, the incidence and prevalence of CLD continue to grow, posing challenges for healthcare systems globally (9, 10). The relationship between CLD and mental health disorders is complex and bidirectional.

Patients with CLD often experience psychological distress, which can significantly affect their quality of life and overall health outcomes (11). The stress of living with a chronic condition, alongside the social and economic impacts, contribute to the higher prevalence of mental health issues in CLD patients. Moreover, mental health disorders can negatively impact the management and progression of liver disease, creating a vicious circle. Qin et al. reported that 75% CLD patients had mental health problems, and education levels, course of disease, annual hospitalizations, complications, and nursing satisfaction levels were all independent risk factors for the mental health of patients with CLD (12). Duan et al. revealed that patients with hepatitis B virus (HBV)-related acute-on-chronic liver failure and cirrhosis are at a higher risk of depression, and concluded that lower education level, anxiety, poor sleep quality, and greater severity of disease were associated with elevated depression (13). Therefore, mental problems in CLD patients need urgent attention. Most previous studies were conducted on hospitalized patients. Outpatients, navigating their daily lives in a prolonged and chronic disease state, necessitate further exploration into the effects of long-term illness on mental health. Therefore, this study aimed to investigate the mental health status and explore risk factors of patients with CLD in outpatients.

## Method

### Study design

The questionnaire was developed by psychologists, hepatologists and caregivers. All outpatients completed the anonymous questionnaire under the guidance of trained nursing staff. The diagnosis of CLD followed the clinical practice guidelines. 7-tiem generalized anxiety disorder scale (GAD-7), patient health questionnaire-9 (PHQ-9), and Pittsburgh sleep quality index (PSQI) were used to examine anxiety, depression, and sleep quality of patients, respectively.

### Data collection and self-rating mental health scale

Demographic data included sex, age, height, weight, education level, place of residence, smoking, drinking, diabetes, hypertension, chronic kidney disease, malignancy, disease duration, drug therapy or not, and drug use duration. BMI was calculated with formula BMI = weight (kg) divided by height (m^2^). GAD-7 scale is a simple self-rating scale commonly used in primary care settings (14). The scale consists of 7 items. Each entry has a score ranging from 0 to 4 points, depending on the severity of the disease. A total score greater than 4 is considered anxiety. The scale has also been verified in different populations and was proved to have a good efficacy (15, 16). PHQ-9 is a simple and effective self-rating scale for depressive disorder (17). It has good reliability and validity in assisting the diagnosis of depression and evaluating the severity of symptoms (18, 19). The scale consists of 9 items, each of which is scored from 0 to 3, with a score of more than 4 being considered depressed. PSQI contains a total of 15 entries (20). The scale is used to assess the sleep quality of the subjects for nearly 1 month and is based on 19 self-rating questions. The 19 self-rating questions are scored on a scale of 0 to 3, with a score of more than 5 indicating a sleep disorder. PSQI scale was proved to have a good diagnostic accuracy (21, 22).

### Quality control

The questionnaire was designed by psychologists, hepatologists and nursing staff collectively to ensure the rationality of the questionnaire design. The questionnaire was completed under the guidance of the trained nurses. During data processing stage, the outliers were identified, and internal logic judgment was made. For missing values, if a variable was more than 30% missing, multiple imputation was performed. Cronbach’s α was used to test reliability. Kaiser-Meyer-Olkin (KMO) and Bartlett test were used to perform validity analysis.

### Statistical analysis

Continuous variables were presented as the mean ± standard deviation or median with the 25th and 75th percentiles. Categorical variables were presented as frequencies and percentages and. Shapiro-Wilk test was used to verify the normality of variable distribution. Mann-Whitney U tests was used for continuous variables, while the Chi square test was used for comparing categorical variables between different groups. Univariate and multivariate logistic regression analyses were used to evaluate the risk factors affecting mental health. Restricted cubic spline (RCS) was used to examine the potential non-linear associations. Analyses were completed in R (4.2.1), SPSS 27, and Sangerbox (23). *P*<0.05 was considered statistically significant.

## Result

### General characteristics of the study population

**Figure 1:**
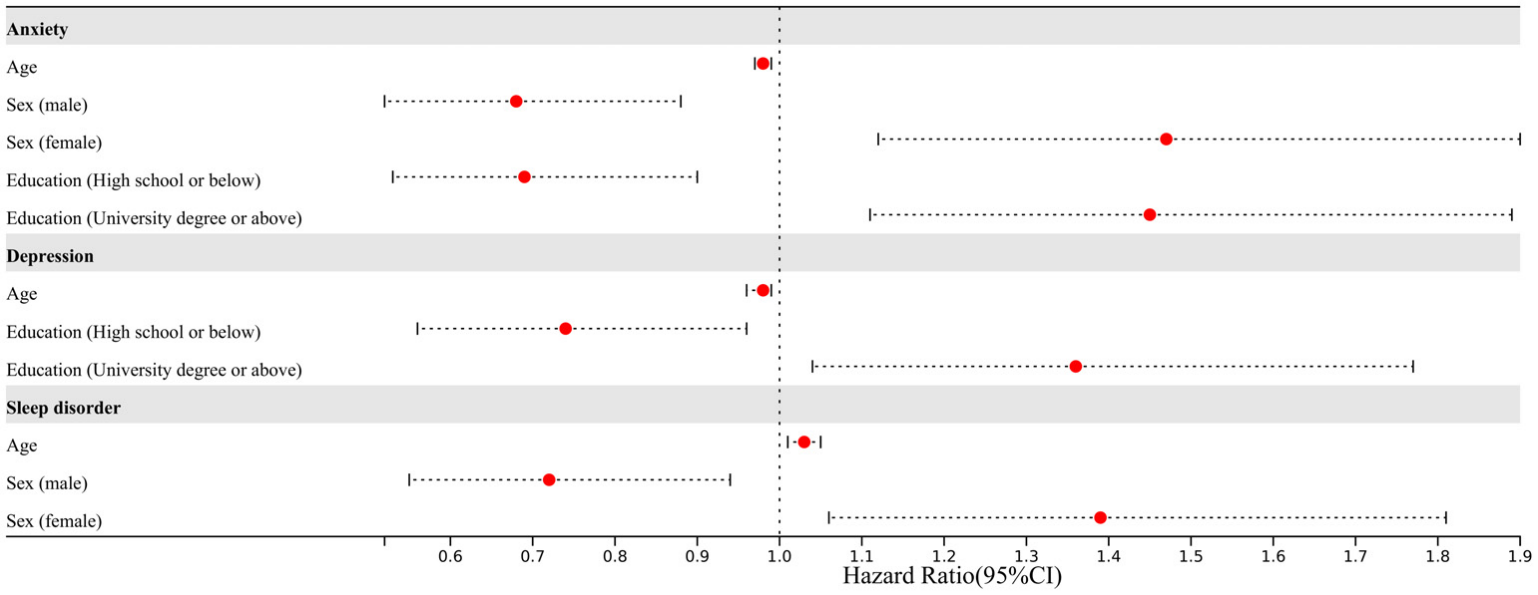
The disease distribution of patients in our study. MRLD, metabolic related liver disease; AIL, autoimmune liver disease.

In total, 1030 questionnaires were collected. Following quality control measures, 1003 questionnaires were suitable for inclusion in analysis. The study encompassed 888 individuals with hepatitis, 124 with advanced liver disease (cirrhosis, liver failure, and liver cancer), 54 with metabolically related liver diseases (such as alcoholic liver disease and non-alcoholic liver disease), 14 with autoimmune liver diseases, and 60 with other types of liver diseases. The reliability and validity of the questionnaire were robust, as demonstrated by Cronbach’s α scores of 0.895, 0.887, and 0.768 and the KMO values of 0.906, 0.916, and 0.852 for GAD-7, PHQ-9, and PSQI respectively. 564 out of 1003 individuals had anxiety, 534 out of 1003 had depression, and 676 out of 1003 had sleep disorders (**Figure 1**).

### Independent risk factors influencing anxiety in patients with CLD

**Figure 2:**
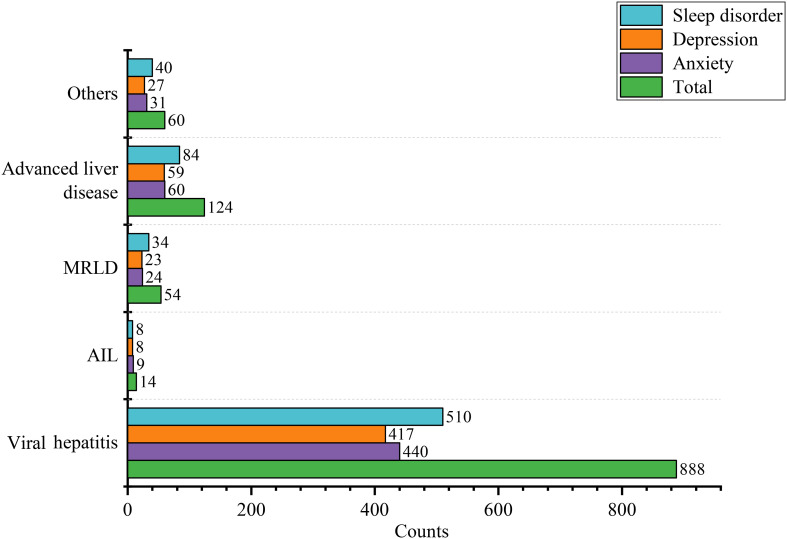
Forest plot of different risk factors of mental disorder after multivariate logistic regression.

**Figure 3:**
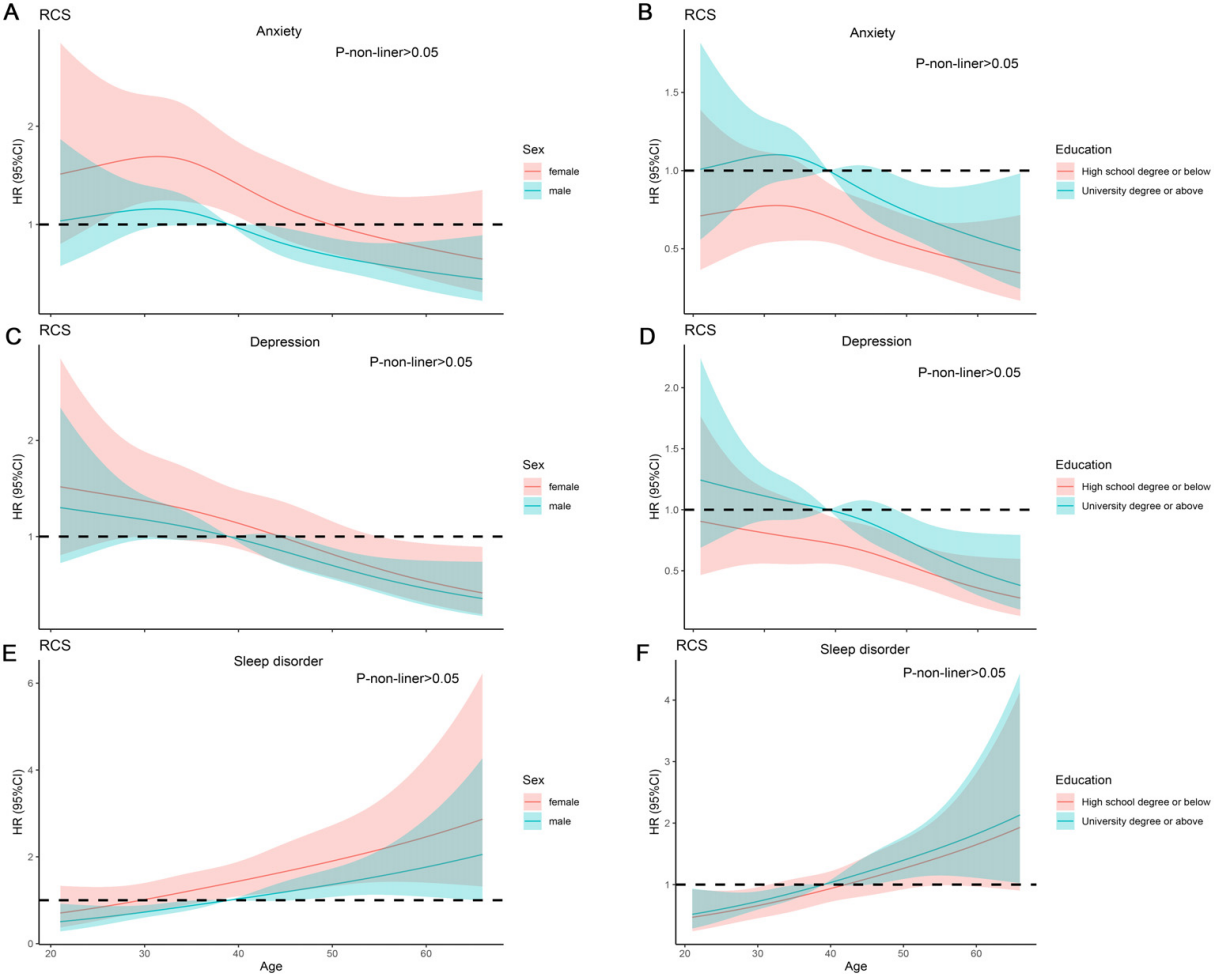
RCS analysis on the association between the age and mental disorder. **(A, B)** RCS curve of the association between the age and anxiety among sex and education. **(C, D)** RCS curve of the association between the age and Depression among sex and education. **(E, F)** RCS curve of the association between the age and sleep disorder among sex and education.

We found that compared to the anxiety-free group, the anxious population differed in gender, age, educational level, hypertension, disease duration, depression, sleep disorders, GAD-7, PHQ-9, and PSQI score (**Table 1**). Subsequently, we conducted subgroup analyses for gender, age, and educational level separately, and we found that the demographic differences were essentially still present (**Supplementary Tables 1**, **4**, **7**). After univariate logistic regression, gender, age, educational level, and hypertension were found to be related to anxiety (**Supplementary Table 10**). Further multivariate logistic regression revealed that age (OR=0.98, 95% CI=0.97-0.99, *P*=0.02) and male (OR=0.68, 95%CI=0.52-0.88, *P*=0.004) were protective factors against anxiety. University education levels (OR=1.45, 95%CI=1.11-1.89, *P*=0.007) were associated with a higher risk of anxiety (**Figure 2**). RCS curve did not observe a nonlinear association between age and anxiety across different sex and education populations (**Figures 3A, B**).

**Table 1:** Demographic characteristics of different mental disorder groups.

Variables	Anxiety			Depression			Sleep disorder		
	No (N=513)	Yes (N=490)	*P*	No (N=533)	Yes (N=470)	*P*	No (N=421)	Yes (N=582)	*P*
Sex, %			**0.005**			0.25			**0.04**
Female	153 (29.8)	188 (38.4)		172 (32.3)	169 (36.0)		127 (30.2)	214 (36.8)	
Male	360 (70.2)	302 (61.6)		361 (67.7)	301 (64.0)		294 (69.8)	368 (63.2)	
Age [Median, IQR]	40 (32,48)	37 (31,46)	**<0.001**	40 (33,48)	36 (31,45.75)	**<0.001**	36 (30,45)	40 (32.25,48)	**<0.001**
BMI [Median, IQR]	20.7 (22.49,24.65)	22.49 (20.13,24.22)	0.08	22.58 (20.72,24.62)	22.37 (20.17,24.22)	0.09	22.27 (20.32,24.46)	22.67 (20.57,24.56)	0.54
Education, %			**<0.001**			**<0.001**			0.39
High school degree or below	267 (52.0)	198 (40.4)		275 (51.6)	190 (40.4)		188 (44.7)	277 (47.6)	
University degree or above	246 (48.0)	292 (59.6)		258 (48.4)	280 (59.6)		233 (55.3)	305 (52.4)	
Location, %			0.79			0.69			0.59
Rural	181 (35.3)	168 (34.3)		182 (34.1)	167 (35.5)		151 (35.9)	198 (34.0)	
Urban	332 (64.7)	322 (65.7)		351 (65.9)	303 (64.5)		270 (64.1)	384 (66.0)	
Smoking, %			0.95			0.2			1.00
No	480 (93.6)	457 (93.3)		426 (79.9)	359 (76.4)		329 (78.1)	456 (78.4)	
Yes	33 (6.4)	33 (6.7)		107 (20.1)	111 (23.6)		92 (21.9)	126 (21.6)	
Drinking, %			0.95			0.24			0.28
No	480 (93.6)	457 (93.3)		503 (94.4)	434 (92.3)		398 (94.5)	539 (92.6)	
Yes	33 (6.4)	33 (6.7)		30 (5.6)	36 (7.7)		23 (5.5)	43 (7.4)	
HBP, %			**0.04**			0.42			0.11
No	488 (95.1)	479 (97.8)		511 (95.9)	456 (97.0)		411 (97.6)	556 (95.5)	
Yes	25 (4.9)	11 (2.2)		22 (4.1)	14 (3.0)		10 (2.4)	26 (4.5)	
Diabetes, %			0.42			1.00			0.68
No	495(96.5)	478 (97.6)		517 (97.0)	456 (97.0)		410 (97.4)	563 (96.7)	
Yes	18 (3.5)	12 (2.4)		16 (3.0)	14 (3.0)		11 (2.6)	19 (3.3)	
Obesity, %			0.55			0.14			0.34
No	489 (95.3)	462 (94.3)		511(95.9)	440 (93.6)		403 (95.7)	548 (94.2)	
Yes	24 (4.7)	28 (5.7)		22 (4.1)	30 (6.4)		18 (4.3)	34 (5.8)	
Malignancy, %			1			0.88			0.56
No	502 (97.9)	480 (98.0)		521 (97.7)	461(98.1)		414 (98.3)	568 (97.6)	
Yes	11 (2.1)	10 (2.0)		12 (2.3)	9 (1.9)		7 (1.7)	14 (2.4)	
CKD, %			0.92			1.00			0.07
No	505 (98.4)	481 (98.2)		524 (98.3)	462(98.3)		418 (99.3)	568 (97.6)	
Yes	8 (1.6)	9 (1.8)		9 (1.7)	8 (1.7)		3 (0.7)	14 (2.4)	
Disease duration, %			**0.02**			**0.01**			**0.04**
<3years	84 (16.4)	69 (14.1)		86 (16.1)	67 (14.3)		56 (13.3)	97 (16.7)	
3-5years	45 (8.8)	60 (12.2)		47 (8.8)	58 (12.3)		45 (10.7)	60 (10.3)	
6-10years	91 (17.7)	59 (12.0)		90 (16.9)	60 (12.8)		79 (18.8)	71 (12.2)	
10-20years	132 (25.7)	151(30.8)		132 (24.8)	151 (32.1)		112 (26.6)	171 (29.4)	
20 years+	151 (30.8)	161 (31.4)		178 (33.4)	134 (28.5)		129 (30.6)	183 (31.4)	
Drug therapy, %			0.79			**0.04**			0.50
No	116 (22.6)	107 (21.8)		132 (24.8)	91 (19.4)		98 (23.3)	125 (21.5)	
Yes	397 (77.4)	383 (78.2)		401 (75.2)	379 (80.6)		323 (76.7)	457 (78.5)	
Drug use duration, %			0.24			0.17			0.50
<6months	81 (15.8)	78 (15.9)		77 (14.4)	82 (17.4)		61 (14.5)	98 (16.8)	
6months-1year	33(6.4)	44 (9.0)		40 (7.5)	37 (7.9)		35 (8.3)	42 (7.2)	
1-2years	90 (17.5)	79 (16.1)		97 (18.2)	72 (15.3)		78 (18.5)	91 (15.6)	
3-5years	76 (14.8)	92 (18.8)		78 (14.6)	90 (19.1)		66 (15.7)	102 (17.5)	
5-10years	74 (14.4)	62 (12.7)		72 (13.5)	64 (13.6)		59 (14.0)	77 (13.2)	
>10years	43 (8.4)	28 (5.7)		37 (6.9)	34 (7.2)		24 (5.7)	47 (8.1)	
No	116 (22.6)	107 (21.8)		132 (24.8)	91 (19.4)		98 (23.3)	125 (21.5)	
GAD-7 [Median, IQR]	1(0,3)	7 (6,10)	**<0.001**	2 (0,4)	7 (5,10)	**<0.001**	2 (0,5)	6 (3,9)	**<0.001**
PHQ-9 [Median, IQR]	2 (0,4)	8 (5,11)	**<0.001**	2(0,3)	8 (6,11)	**<0.001**	2 (0,4)	6 (3,9)	**<0.001**
PSQI [Median, IQR]	2 (3,7)	8 (5,10)	**<0.001**	5 (3,7)	8 (6,11)	**<0.001**	3 (3,4)	8 (7,11)	**<0.001**
Depression, %			**<0.001**			–			**<0.001**
No	427 (83.2)	106 (21.6)		–	–		297 (70.5)	216 (37.1)	
Yes	86 (16.8)	384 (78.4)		–	–		124 (29.5)	366 (62.9)	
Sleep disorder, %			**<0.001**			**<0.001**			–
No	297 (57.9)	124 (25.3)		329 (61.7)	92 (19.6)		–	–	
Yes	216 (42.1)	366 (74.7)		204 (38.3)	378 (80.4)		–	–	
Anxiety, %			–			**<0.001**			**<0.001**
No	–	–		427 (80.1)	86 (18.3)		297 (70.5)	216 (37.1)	
Yes	–	–		106(19.9)	384 (81.7)		124 (29.5)	366 (62.9)	

IQR, inter quartile range; HBP, high blood pressure; CKD, chronic kidney disease; GAD-7,7-tiem Generalized Anxiety Disorder Scale; PHQ-9, Patient Health Questionnaire-9; PSQI, Pittsburgh sleep quality index. Bold values emphasize differences in indicators.

### Independent risk factors affecting depression in patients with CLD

We found that compared to the depress-free group, the depressed population differed in gender, educational level, disease duration, drug therapy, anxiety, sleep disorder, GAD-7, PHQ-9, and PSQI score (**Table 1**). Subgroup analysis indicated that demographic disparities remained unchanged (**Supplementary Tables 2**, **5**, **8**). After univariate logistic regression, age and educational level were identified as factors associated with depression. Further multivariate logistic regression revealed that age (OR=0.98, 95%CI=0.96-0.99, *P*<0.001) was a protective factor against depression (**Supplementary Table 10**). University education levels (OR=1.36, 95%CI=1.04-1.77, *P*=0.02) were associated with a higher risk of depression (**Figure 2**). RCS curve did not observe nonlinear association between age and depression across different sex and education populations (**Figures 3C, D**).

### Independent risk factors impacting sleep disorder in patients with CLD

We discovered that compared to the control group, the population with sleep disorders differed in gender, age, duration of illness, anxiety, depression, GAD-7, PHQ-9, and PSQI score (**Table 1**). Subgroup analyses demonstrated that the demographic characteristics remained unchanged (**Supplementary Tables 3**, **6**, **9**). After univariate logistic regression, gender and age were determined to be factors linked to sleep disorders (**Supplementary Table 10**). Further multivariate logistic regression revealed that age was associated with a higher risk of sleep disorders (OR=1.03, 95%CI: 1.01-1.05, *P* < 0.001), and male was less likely to have sleep disorders (OR=0.72, 95%CI: 0.55-0.94, *P* = 0.02) (**Figure 2**). RCS curve did not observe a nonlinear association between age and sleep disorder among sex and education populations (**Figures 3E, F**).

## Discussion

CLD is a major health issue, the long-term course of the disease can damage patients’ mental health. In CLD population, mental health issues are often overlooked, affecting the effectiveness of disease treatment, and prolonging the duration of the illness. In this study, we investigated the psychological condition of the CLD population through a questionnaire survey. We found that nearly half of the CLD patients suffer from anxiety, depression, and sleep disorders. After multivariate logistic regression, we found that age, gender, and educational level are independent risk factors for mental disorders. RCS analysis revealed that the relationship between age and mental disorders is not nonlinear in different populations.

The relationship between age and anxiety is complex. Some studies suggested that older people suffer from at least one chronic disease and face greater health issues than younger individuals (24). However, younger people also encountered more social pressures, including socializing, academics, work, and balancing family life (25, 26). Anxiety sensitivity and an intolerance of uncertainty may make both older and younger individuals more prone to anxiety (24). As for the relationship between age and depression, some research indicated that the incidence of depression decreases with age (27), while other reports have shown a U-shaped relationship between age and depression (28, 29). In our study, we found a negative correlation between age and both depression and anxiety, with younger individuals with CLD being more susceptible to anxiety and depression. Young people’s extensive internet access leads to more online health information seeking, potentially causing unnecessary anxiety. Older adults might have identified their health issues earlier but faced restricted treatment options and access, leading to extended periods without medication or regular check-ups. This reduced focus on disease progression could lower mental health concerns.

Studies reveal that the female population was more susceptible to depression, with a male to female ratio of 2:1 (30), but other studies suggested that there are no gender differences in the incidence of depression in children. A recent meta-analysis showed that gender differences in the incidence of depression peak during adolescence, but decline and stabilize after adulthood (31). Repeated reductions in estrogen can interfere with the ability of estrogen to neutralize the release of glucocorticoids when feeling stressed, making women more susceptible to stress and thus at risk of anxiety and depression (32, 33). Many traits of women, including changes occurring in the biological lifecycle such as menstruation, pregnancy, and menopause, increase the risk of sleep disorders (34). Sex hormones may play a role in sleep regulation and arousal (35, 36). Studies suggested that due to differences in brain chemicals and the stress response system, women may be physiologically more prone to anxiety disorders (37). For example, differences in the neurotransmitter serotonin are considered a factor in the higher incidence of anxiety disorders in women (38, 39).

Research showed that the education level was negatively correlated with mental health problems in CLD patients (40), and protective effects were higher among women (41). Some research found there was not protective role of education in mental health (42). In our study, we found that having a university education or higher increase the risk of anxiety and depression in patients with CLD. Their propensity for analytical thinking, perfectionism, and extensive mental activity of university education increased sleep disorder risk (43).

There are several limitations in our article. Firstly, our samples came from a single center, and multi-center data is needed to assess the relationship between CLD and mental disorders. Secondly, selection bias cannot be excluded Third, more socioeconomic and family support needs to be included in the questionnaire analysis. Fourth, the population included in our study was mostly viral hepatitis patients, which may misevaluate mental health in other liver diseases.

## Conclusion

In summary, we revealed that at least half of the population with CLD suffer from mental disorders. Age, gender, and education level may be independent risk factors for mental illness. Given the high prevalence of mental illness among patients with CLD, the use of screening tools in clinic may identify those at greatest need of attention from a psychologist.

## Data Availability

The raw data supporting the conclusions of this article will be made available by the authors, without undue reservation.
