# Supplementary material for: Mental health disorder in chronic liver disease: a questionnaire survey
Source: Front Psychiatry. 2024 Oct 25;15:1469372. doi: 10.3389/fpsyt.2024.1469372 (PMC11543405; doi:10.3389/fpsyt.2024.1469372)
Supplement: Supplementary file 1 [file Table1.docx]

Supplementary Table 1 Subgroup analysis of chronic liver disease and anxiety stratified by sex.

| Variables | Male | | | Female | | |
| --- | --- | --- | --- | --- | --- | --- |
|  | Anxiety | | | Anxiety | | |
|  | No  (N=360) | Yes  (N=302) | *P* | No  (N=153) | Yes  (N=188) | *P* |
| Age  [Median, IQR] | 40 (33,48) | 37 (31,45) | **<0.001** | 39 (31,47) | 37 (30,47) | 0.36 |
| BMI  [Median, IQR] | 23.2  (21.2, 25.4) | 23.1  (21.0, 24.7) | 0.25 | 21.1  (20.0, 23.1) | 21.3  (19.5, 23.3) | 0.75 |
| Education, % |  |  | **<0.001** |  |  | 0.19 |
| High school degree or below | 186 (51.7) | 113 (37.4) |  | 81 (52.9) | 85 (45.2) |  |
| University degree or above | 174 (48.3) | 189 (62.6) |  | 72 (47.1) | 103 (54.8) |  |
| Location, % |  |  | 0.31 |  |  | 0.35 |
| Rural | 134 (37.2) | 100 (33.1) |  | 47 (30.7) | 68 (36.2) |  |
| Urban | 226 (62.8) | 202 (66.9) |  | 106 (69.3) | 120 (63.8) |  |
| Smoking, % |  |  | 0.56 |  |  | 1.00 |
| No | 253 (70.3) | 205 (67.9) |  | 147 (96.1) | 180 (95.7) |  |
| Yes | 107 (29.7) | 97 (32.1) |  | 6 (3.9) | 8 (4.3) |  |
| Drinking, % |  |  | 0.97 |  |  | 0.32 |
| No | 328 (91.1) | 274 (90.7) |  | 152 (99.3) | 183 (97.3) |  |
| Yes | 32 (8.9) | 28 (9.3) |  | 1 (0.7) | 5 (2.7) |  |
| HBP, % |  |  | **0.02** |  |  | 0.95 |
| No | 341 (94.7) | 297 (98.3) |  | 147 (96.1) | 182 (96.8) |  |
| Yes | 19 (5.3) | 5 (1.7) |  | 6 (3.9) | 6 (3.2) |  |
| Diabetes, % |  |  | 0.31 |  |  | 1.00 |
| No | 347 (96.4) | 296 (98.0) |  | 148 (96.7) | 182 (96.8) |  |
| Yes | 13 (3.6) | 6 (2.0) |  | 5 (3.3) | 6 (3.2) |  |
| Obesity, % |  |  | 0.22 |  |  | 0.75 |
| No | 341 (94.7) | 278 (92.1) |  | 148 (96.7) | 184 (97.9) |  |
| Yes | 19 (5.3) | 24 (7.9) |  | 5 (3.3) | 4 (2.1) |  |
| Malignancy, % |  |  | 0.97 |  |  | 1.00 |
| No | 354 (98.3) | 298 (98.7) |  | 148 (96.7) | 182 (96.8) |  |
| Yes | 6 (1.7) | 4 (1.3) |  | 5 (3.3) | 6 (3.2) |  |
| CKD, % |  |  | 1.00 |  |  | 0.95 |
| No | 355 (98.6) | 298 (98.7) |  | 150 (98.0) | 183 (97.3) |  |
| Yes | 5 (1.4) | 4 (1.3) |  | 3 (2.0) | 5 (2.7) |  |
| Disease duration, % |  |  | **0.03** |  |  | 0.52 |
| <3years | 50 (13.9) | 37 (12.3) |  | 34 (22.2) | 32 (17.0) |  |
| 3-5years | 31(8.6) | 34 (11.3) |  | 14(9.2) | 26 (13.8) |  |
| 6-10years | 70 (19.4) | 37 (12.3) |  | 21 (13.7) | 22 (11.7) |  |
| 10-20years | 92 (25.6) | 102 (33.8) |  | 40 (26.1) | 49 (26.1) |  |
| 20 years+ | 117 (32.5) | 92 (30.5) |  | 44 (28.8) | 59 (31.4) |  |
| Drug therapy, % |  |  | 0.25 |  |  | 0.60 |
| No | 76 (21.1) | 53 (17.5) |  | 40 (26.1) | 54 (28.7) |  |
| Yes | 284 (78.9) | 249 (82.5) |  | 113 (73.9) | 134 (71.3) |  |
| Drug use duration, % |  |  | 0.29 |  |  | 0.23 |
| <6months | 54 (15.0) | 52 (17.2) |  | 27 (17.6) | 26 (13.8) |  |
| 6months-1year | 22(6.1) | 29(9.6) |  | 11(7.2) | 15(8.0) |  |
| 1-2years | 56 (15.6) | 51 (16.9) |  | 34 (22.2) | 28 (14.9) |  |
| 3-5years | 57 (15.8) | 52 (17.2) |  | 19 (12.4) | 40 (21.3) |  |
| 5-10years | 56 (15.6) | 44 (14.6) |  | 18 (11.8) | 18 (9.6) |  |
| >10years | 39 (10.8) | 21 (7.0) |  | 4 (2.6) | 7 (3.7) |  |
| No | 76 (21.1) | 53 (17.5) |  | 40 (26.1) | 54 (28.7) |  |
| GAD-7  [Median, IQR] | 1 (0,3) | 7 (6,10) | **<0.001** | 2 (0,3) | 8 (6,10) | **<0.001** |
| PHQ-9  [Median, IQR] | 2 (0,4) | 8 (5,11) | **<0.001** | 2 (0,3) | 7 (5,11) | **<0.001** |
| PSQI  [Median, IQR] | 5 (3,7) | 8 (5,10) | **<0.001** | 5 (3,8) | 8 (6,11) | **<0.001** |
| Depression, % |  |  | **<0.001** |  |  | - |
| No | 300 (83.3) | 61 (20.2) |  | 127 (83.0) | 45 (23.9) |  |
| Yes | 60 (16.7) | 241 (79.8) |  | 26 (17.0) | 143 (76.1) |  |
| Sleep disorder, % |  |  | **<0.001** |  |  | **<0.001** |
| No | 212 (58.9) | 82 (27.2) |  | 85 (55.6) | 42 (22.3) |  |
| Yes | 148 (41.1) | 220 (72.8) |  | 68 (44.4) | 146 (77.7) |  |

Note: IQR: inter quartile range; HBP: high blood pressure; CKD: chronic kidney disease; GAD-7,7-tiem

Generalized Anxiety Disorder Scale; PHQ-9, Patient Health Questionnaire-9; PSQI, Pittsburgh sleep quality

index.
