# Supplementary material for: Mental health disorder in chronic liver disease: a questionnaire survey
Source: Front Psychiatry. 2024 Oct 25;15:1469372. doi: 10.3389/fpsyt.2024.1469372 (PMC11543405; doi:10.3389/fpsyt.2024.1469372)
Supplement: Supplementary file 2 [file Table2.docx]

Supplementary Table 2 Subgroup analysis of chronic liver disease and depression stratified by sex.

| Variables | Male | | | Female | | |
| --- | --- | --- | --- | --- | --- | --- |
|  | Depression | | | Depression | | |
|  | No  (N=361) | Yes  (N=301) | *P* | No  (N=172) | Yes  (N=169) | *P* |
| Age  [Median, IQR] | 40 (33,48) | 37 (31,36) | **<0.001** | 39 (31.8, 48) | 36 (29,45) | **0.02** |
| BMI  [Median, IQR] | 23.2 (21.3,25.3) | 23.1 (21.1,24.8) | 0.17 | 21.4 (20,23.2) | 21 (19.6,23.2) | 0.47 |
| Education, % |  |  | **<0.001** |  |  | 0.41 |
| High school degree or below | 187 (51.8) | 112 (37.2) |  | 88 (51.2) | 78 (46.2) |  |
| University degree or above | 174 (48.2) | 189 (62.8) |  | 84 (48.8) | 91 (53.8) |  |
| Location, % |  |  | 0.34 |  |  | **0.02** |
| Rural | 134 (37.1) | 100 (33.2) |  | 48 (27.9) | 67 (39.6) |  |
| Urban | 227 (62.9) | 201 (66.8) |  | 124(72.1) | 102 (60.4) |  |
| Smoking, % |  |  | 0.25 |  |  | 0.05 |
| No | 257 (71.2) | 201 (66.8) |  | 169 (98.3) | 158 (93.5) |  |
| Yes | 104 (28.8) | 100 (33.2) |  | 3 (1.7) | 11 (6.5) |  |
| Drinking, % |  |  | 0.55 |  |  | **0.03** |
| No | 331 (91.7) | 271 (90.0) |  | 172 (100) | 163 (96.4) |  |
| Yes | 30 (8.3) | 30 (10.0) |  | 0 (0) | 6 (3.6) |  |
| HBP, |  |  | 0.32 |  |  | 1 |
| No | 345 (95.6) | 293 (97.3) |  | 166 (96.5) | 163 (96.4) |  |
| Yes | 16 (4.4) | 8 (2.7) |  | 6 (3.5) | 6 (3.6) |  |
| Diabetes, % |  |  | 0.60 |  |  | 0.52 |
| No | 349 (96.7) | 294 (97.7) |  | 168 (97.7) | 162 (95.9) |  |
| Yes | 12 (3.3) | 7 (2.3) |  | 4 (2.3) | 7 (4.1) |  |
| Obesity, % |  |  | 0.12 |  |  | 0.98 |
| No | 343 (95.0) | 276 (91.7) |  | 168 (97.7) | 164 (97.0) |  |
| Yes | 18 (5.0) | 25 (8.3) |  | 4 (2.3) | 5 (3.0) |  |
| Malignancy, % |  |  | 0.98 |  |  | 1 |
| No | 355 (98.3) | 297 (98.7) |  | 166 (96.5) | 164 (97.0) |  |
| Yes | 6 (1.7) | 4 (1.3) |  | 6 (3.5) | 5 (3.0) |  |
| CKD, % |  |  | 0.69 |  |  | 0.70 |
| No | 355 (98.3) | 298 (99.0) |  | 169 (98.3) | 164 (97.0) |  |
| Yes | 6 (1.7) | 3 (1.0) |  | 3 (1.7) | 5 (3.0) |  |
| Disease duration, % |  |  | **0.02** |  |  | 0.46 |
| <3years | 48 (13.3) | 39 (13.0) |  | 38 (22.1) | 28 (16.6) |  |
| 3-5years | 31 (8.6) | 34 (11.3) |  | 16 (9.3) | 24 (14.2) |  |
| 6-10years | 68 (18.8) | 39 (13.0) |  | 22 (12.8) | 21 (12.4) |  |
| 10-20years | 90 (24.9) | 104 (34.6) |  | 42 (24.4) | 47 (27.8) |  |
| 20 years+ | 124 (34.3) | 85 (28.2) |  | 54 (31.4) | 49 (29.0) |  |
| Drug therapy, % |  |  | **0.01** |  |  | 0.70 |
| No | 83 (23.0) | 46 (15.3) |  | 49 (28.5) | 45 (26.6) |  |
| Yes | 278 (77.0) | 255 (84.7) |  | 123 (71.5) | 124 (73.4) |  |
| Drug use duration, % |  |  | 0.17 |  |  | 0.11 |
| <6months | 49 (13.6) | 57 (18.9) |  | 28 (16.3) | 25 (14.8) |  |
| 6months-1year | 25 (6.9) | 26 (8.6) |  | 15 (8.7) | 11 (6.5) |  |
| 1-2 years | 59 (16.3) | 48 (15.9) |  | 38 (22.1) | 24 (14.2) |  |
| 3-5 years | 56 (15.5) | 53 (17.6) |  | 22 (12.8) | 37 (21.9) |  |
| 5-10 years | 55 (15.2) | 45 (15.0) |  | 17 (9.9) | 19 (11.2) |  |
| >10 years | 34 (9.4) | 26 (8.6) |  | 3 (1.7) | 8 (4.7) |  |
| No | 83 (23.0) | 46 (15.3) |  | 49 (28.5) | 45 (26.6) |  |
| GAD-7  [Median, IQR] | 2 (0,4) | 7 (5,10) | **<0.001** | 2 (1,5) | 8 (6,11) | **<0.001** |
| PHQ-9  [Median, IQR] | 2 (0,3) | 8 (6,11) | **<0.001** | 2 (1,5) | 8 (6,11) | **<0.001** |
| PSQI  [Median, IQR] | 4 (3,6) | 8 (6,10) | **<0.001** | 5 (3,7.25) | 8 (6,12) | **<0.001** |
| Anxiety, % |  |  | **<0.001** |  |  | **<0.001** |
| No | 300 (83.1) | 60 (19.9) |  | 127 (73.8) | 26 (15.4) |  |
| Yes | 61 (16.9) | 241 (80.1) |  | 45 (26.2) | 143 (84.6) |  |
| Sleep disorder, % |  |  | **<0.001** |  |  | **<0.001** |
| No | 234 (64.8) | 60 (19.9) |  | 95 (55.2) | 32 (18.9) |  |
| Yes | 127 (35.2) | 241 (80.1) |  | 77 (44.8) | 137 (81.1) |  |

Note: IQR: inter quartile range; HBP: high blood pressure; CKD: chronic kidney disease; GAD-7,7-tiem

Generalized Anxiety Disorder Scale; PHQ-9, Patient Health Questionnaire-9; PSQI, Pittsburgh sleep quality

index.
