# Supplementary material for: Mental health disorder in chronic liver disease: a questionnaire survey
Source: Front Psychiatry. 2024 Oct 25;15:1469372. doi: 10.3389/fpsyt.2024.1469372 (PMC11543405; doi:10.3389/fpsyt.2024.1469372)
Supplement: Supplementary file 3 [file Table3.docx]

Supplementary Table 3 Subgroup analysis of chronic liver disease and sleep disorder stratified by sex.

| Variables | Male | | | Female | | |
| --- | --- | --- | --- | --- | --- | --- |
|  | Sleep disorder | | | Sleep disorder | | |
|  | No  (N=294) | Yes  (N=368) | *P* | No  (N=127) | Yes  (N=214) | *P* |
| Age  [Median, IQR] | 37 (31, 46) | 40 (33,48) | **0.002** | 34 (29,42) | 40 (32,49) | **<0.001** |
| BMI  [Median, IQR] | 23.1 (21,24.8) | 23.4 (21.2,25.2) | 0.45 | 20.8 (19.6,22.5) | 21.5 (19.8,23.3) | 0.21 |
| Education, % |  |  | 0.96 |  |  | 0.23 |
| High school degree or below | 132 (44.9) | 167 (45.4) |  | 56 (44.1) | 110 (51.4) |  |
| University degree or above | 162 (55.1) | 201 (54.6) |  | 71 (55.9) | 104 (48.6) |  |
| Location, % |  |  | 0.45 |  |  | 0.94 |
| Rural | 109 (37.1) | 125 (34.0) |  | 42 (33.1) | 73 (34.1) |  |
| Urban | 185 (62.9) | 243 (66.0) |  | 85 (66.9) | 141 (65.9) |  |
| Smoking, % |  |  | 0.85 |  |  | 0.33 |
| No | 205 (69.7) | 253 (68.8) |  | 124 (97.6) | 203 (94.9) |  |
| Yes | 89 (30.3) | 115 (31.3) |  | 3 (2.4) | 11 (5.1) |  |
| Drinking, % |  |  | 0.26 |  |  | 0.53 |
| No | 272 (92.5) | 330 (89.7) |  | 126 (99.2) | 209 (97.7) |  |
| Yes | 22 (7.5) | 38 (10.3) |  | 1 (0.8) | 5 (2.3) |  |
| HBP, % |  |  | 0.36 |  |  | 0.23 |
| No | 286 (97.3) | 352 (95.7) |  | 125 (98.4) | 204 (95.3) |  |
| Yes | 8 (2.7) | 16 (4.3) |  | 2 (1.6) | 10 (4.7) |  |
| Diabetes, % |  |  | 0.98 |  |  | 0.31 |
| No | 285 (96.9) | 358 (97.3) |  | 125 (98.4) | 205 (95.8) |  |
| Yes | 9 (3.1) | 10 (2.7) |  | 2 (1.6) | 9 (4.2) |  |
| Obesity, % |  |  | 0.25 |  |  | 1.00 |
| No | 279 (94.9) | 340 (92.4) |  | 124 (97.6) | 208 (97.2) |  |
| Yes | 15 (5.1) | 28 (7.6) |  | 3 (2.4) | 6 (2.8) |  |
| Malignancy, % |  |  | 0.55 |  |  | 1.00 |
| No | 291 (99.0) | 361 (98.1) |  | 123 (96.9) | 207 (96.7) |  |
| Yes | 3 (1.0) | 7 (1.9) |  | 4 (3.1) | 7 (3.3) |  |
| CKD, % |  |  | 0.73 |  |  | 0.06 |
| No | 291 (99.0) | 362 (98.4) |  | 127 (100) | 206 (96.3) |  |
| Yes | 3 (1.0) | 6 (1.6) |  | 0 (0) | 8 (3.7) |  |
| Disease duration, % |  |  | **0.04** |  |  | 0.33 |
| <3years | 37 (12.6) | 50 (13.6) |  | 19 (15.0) | 47 (22.0) |  |
| 3-5years | 27 (9.2) | 38 (10.3) |  | 18 (14.2) | 22 (10.3) |  |
| 6-10years | 61 (20.7) | 46 (12.5) |  | 18 (14.2) | 25 (11.7) |  |
| 10-20years | 75 (25.5) | 119 (32.3) |  | 37 (29.1) | 52 (24.3) |  |
| 20 years+ | 94 (32.0) | 115 (31.3) |  | 35 (27.6) | 68 (31.8) |  |
| Drug therapy, % |  |  | 0.59 |  |  | 0.45 |
| No | 60 (20.4) | 69 (18.7) |  | 38 (29.9) | 56 (26.2) |  |
| Yes | 234 (79.6) | 299 (81.3) |  | 89 (70.1) | 158 (73.8) |  |
| Drug use duration, % |  |  | 0.30 |  |  | 0.92 |
| <6months | 44 (15.0) | 62 (16.8) |  | 17 (13.4) | 36 (16.8) |  |
| 6months-1year | 26 (8.8) | 25 (6.8) |  | 9 (7.1) | 17 (7.9) |  |
| 1-2years | 53 (18.0) | 54 (14.7) |  | 25 (19.7) | 37 (17.3) |  |
| 3-5years | 45 (15.3) | 64 (17.4) |  | 21 (16.5) | 38 (17.8) |  |
| 5-10years | 47 (16.0) | 53 (14.4) |  | 12 (9.4) | 24 (11.2) |  |
| >10years | 19 (6.5) | 41 (11.1) |  | 5 (3.9) | 6 (2.8) |  |
| No | 60 (20.4) | 69 (18.8) |  | 38 (29.9) | 56 (26.2) |  |
| GAD-7  [Median, IQR] | 2 (0,5) | 6 (3,8) | **<0.001** | 3 (1,5) | 7 (4,9) | **<0.001** |
| PHQ-9  [Median, IQR] | 2 (0,4) | 3 (6,9) | **<0.001** | 2 (0,4.5) | 6 (3,9.75) | **<0.001** |
| PSQI  [Median, IQR] | 3 (3,4) | 8 (7,10.3) | **<0.001** | 3 (3,4) | 9 (7,11) | **<0.001** |
| Anxiety, % |  |  | **<0.001** |  |  | **<0.001** |
| No | 212 (72.1) | 148 (40.2) |  | 85 (66.9) | 68 (31.8) |  |
| Yes | 82 (27.9) | 220 (59.8) |  | 42 (33.1) | 146 (68.2) |  |
| Depression, % |  |  | **<0.001** |  |  | **<0.001** |
| No | 234 (79.6) | 127 (34.5) |  | 95 (74.8) | 77 (36.0) |  |
| Yes | 60 (20.4) | 241 (65.5) |  | 32 (25.2) | 137 (64.0) |  |

Note: IQR: inter quartile range; HBP: high blood pressure; CKD: chronic kidney disease; GAD-7,7-tiem

Generalized Anxiety Disorder Scale; PHQ-9, Patient Health Questionnaire-9; PSQI, Pittsburgh sleep quality

index.
