# Supplementary material for: Mental health disorder in chronic liver disease: a questionnaire survey
Source: Front Psychiatry. 2024 Oct 25;15:1469372. doi: 10.3389/fpsyt.2024.1469372 (PMC11543405; doi:10.3389/fpsyt.2024.1469372)
Supplement: Supplementary file 4 [file Table4.docx]

Supplementary Table 4 Subgroup analysis of chronic liver disease and anxiety stratified by age.

| Variables | Age above median | | | Age below median | | |
| --- | --- | --- | --- | --- | --- | --- |
|  | Anxiety | | | Anxiety | | |
|  | No  (N=286) | Yes  (N=216) | *P* | No  (N=227) | Yes  (N=274) | *P* |
| Sex, % |  |  | **0.02** |  |  | 0.19 |
| Female | 79 (27.6) | 82 (38.0) |  | 74 (32.6) | 106 (38.7) |  |
| Male | 207 (72.4) | 134 (62.0) |  | 153 (67.4) | 168 (61.3) |  |
| BMI  [Median, IQR] | 23.1 (21.3,24.9) | 23.2 (21.0,24.5) | 0.65 | 22.0 (20.1,24.1) | 21.8 (19.7,24.1) | 0.27 |
| Education, % |  |  | 0.15 |  |  | **0.04** |
| High school degree or below | 190 (66.4) | 129 (59.7) |  | 77 (33.9) | 69 (25.2) |  |
| University degree or above | 96 (33.6) | 87 (40.3) |  | 150 (66.1) | 205 (74.8) |  |
| Location, % |  |  | 0.42 |  |  | 0.97 |
| Rural | 97 (33.9) | 65 (30.1) |  | 84 (37.0) | 103 (37.6) |  |
| Urban | 189 (66.1) | 151 (69.9) |  | 143 (63.0) | 171 (62.4) |  |
| Smoking, % |  |  | 0.96 |  |  | 1.00 |
| No | 222 (77.6) | 169 (78.2) |  | 178 (78.4) | 216 (78.8) |  |
| Yes | 64 (22.4) | 47 (21.8) |  | 49 (21.6) | 58 (21.2) |  |
| Drinking, % |  |  | 0.89 |  |  | 0.53 |
| No | 264 (92.3) | 201 (93.1) |  | 216 (95.2) | 256 (93.4) |  |
| Yes | 22 (7.7) | 15 (6.9) |  | 11 (4.8) | 18 (6.6) |  |
| HBP, % |  |  | 0.29 |  |  | 0.27 |
| No | 265 (92.7) | 206 (95.4) |  | 223 (98.2) | 273 (99.6) |  |
| Yes | 21 (7.3) | 10 (4.6) |  | 4 (1.8) | 1 (0.4) |  |
| Diabetes, % |  |  | 0.55 |  |  | 1.00 |
| No | 271 (94.8) | 208 (96.3) |  | 224 (98.7) | 270 (98.5) |  |
| Yes | 15 (5.2) | 8 (3.7) |  | 3 (1.3) | 4 (1.5) |  |
| Obesity, % |  |  | 1.00 |  |  | 0.45 |
| No | 271 (94.8) | 204 (94.4) |  | 218 (96.0) | 258 (94.2) |  |
| Yes | 15 (5.2) | 12 (5.6) |  | 9 (4.0) | 16 (5.8) |  |
| Malignancy, % |  |  | 0.84 |  |  | 0.49 |
| No | 276 (96.5) | 210 (97.2) |  | 226 (99.6) | 270 (98.5) |  |
| Yes | 10 (3.5) | 6 (2.8) |  | 1 (0.4) | 4 (1.5) |  |
| CKD, % |  |  | 0.28 |  |  | 0.40 |
| No | 280 (97.9) | 207 (95.8) |  | 225 (99.1) | 274 (100) |  |
| Yes | 6 (2.1) | 9 (4.2) |  | 2 (0.9) | 0 (0) |  |
| Disease duration, % |  |  | 0.08 |  |  | 0.40 |
| <3years | 51 (17.8) | 36 (16.7) |  | 33 (14.5) | 33 (12.0) |  |
| 3-5years | 21 (7.3) | 19 (8.8) |  | 24 (10.6) | 41 (15.0) |  |
| 6-10years | 49 (17.1) | 20 (9.3) |  | 42 (18.5) | 39 (14.2) |  |
| 10-20years | 50 (17.5) | 51 (23.6) |  | 82 (36.1) | 100 (36.5) |  |
| 20 years+ | 115 (40.2) | 90 (41.7) |  | 46 (20.3) | 61 (22.3) |  |
| Drug therapy, % |  |  | 0.16 |  |  | **0.03** |
| No | 42 (14.7) | 42 (19.4) |  | 74 (32.6) | 65 (23.7) |  |
| Yes | 244 (85.3) | 174 (80.6) |  | 153 (67.4) | 209 (76.3) |  |
| Drug use duration, % |  |  | 0.27 |  |  | 0.08 |
| <6months | 45 (15.7) | 31 (14.4) |  | 36 (15.9) | 47 (17.2) |  |
| 6months-1year | 19 (6.6) | 18 (8.3) |  | 14 (6.2) | 26 (9.5) |  |
| 1-2years | 50 (17.5) | 42 (19.4) |  | 40 (17.6) | 37 (13.5) |  |
| 3-5years | 50 (17.5) | 43 (19.9) |  | 26 (11.5) | 49 (17.9) |  |
| 5-10years | 48 (16.8) | 23 (10.6) |  | 26 (11.5) | 39 (14.2) |  |
| >10years | 32 (11.2) | 17 (7.9) |  | 11 (4.8) | 11 (4.0) |  |
| No | 42 (14.7) | 42 (19.4) |  | 74 (32.6) | 65 (23.7) |  |
| GAD-7  [Median, IQR] | 1 (0,3) | 8 (6,10) | **<0.001** | 2 (0,3) | 7 (6,10) | **<0.001** |
| PHQ-9  [Median, IQR] | 2 (0,3) | 7 (4,11) | **<0.001** | 2 (0,4) | 8 (5,11) | **<0.001** |
| PSQI  [Median, IQR] | 6 (3,8) | 9 (7,12) | **<0.001** | 4 (3,6) | 7 (5,9) | **<0.001** |
| Depression, % |  |  | **<0.001** |  |  | **<0.001** |
| No | 241 (84.3) | 57 (26.4) |  | 186 (81.9) | 49 (17.9) |  |
| Yes | 45 (15.7) | 159 (73.6) |  | 41 (18.1) | 225 (82.1) |  |
| Sleep disorder, % |  |  | **<0.001** |  |  | **<0.001** |
| No | 138 (48.3) | 43 (19.9) |  | 159 (70.0) | 81 (29.6) |  |
| Yes | 148 (51.7) | 173 (80.1) |  | 68 (30.0) | 193 (70.4) |  |

Note: IQR: inter quartile range; HBP: high blood pressure; CKD: chronic kidney disease; GAD-7,7-tiem

Generalized Anxiety Disorder Scale; PHQ-9, Patient Health Questionnaire-9; PSQI, Pittsburgh sleep quality

index.
