# Supplementary material for: Mental health disorder in chronic liver disease: a questionnaire survey
Source: Front Psychiatry. 2024 Oct 25;15:1469372. doi: 10.3389/fpsyt.2024.1469372 (PMC11543405; doi:10.3389/fpsyt.2024.1469372)
Supplement: Supplementary file 5 [file Table5.docx]

Supplementary Table 5 Subgroup analysis of chronic liver disease and depression stratified by age.

| Variables | Age above median | | | Age below median | | |
| --- | --- | --- | --- | --- | --- | --- |
|  | Depression | | | Depression | | |
|  | No  (N=298) | Yes  (N=204) | *P* | No  (N=235) | Yes  (N=266) | *P* |
| Sex, % |  |  | 0.32 |  |  | 0.72 |
| Female | 90 (30.2) | 71 (34.8) |  | 82 (34.9) | 98 (36.8) |  |
| Male | 208 (69.8) | 133 (65.2) |  | 153 (65.1) | 168 (63.2) |  |
| BMI  [Median, IQR] | 23.2 (21.3,24.9) | 23.1 (20.7,24.5) | 0.37 | 22.0 (20.0,24.1) | 21.8 (19.8,24.8) | 0.66 |
| Education, % |  |  | 0.13 |  |  | 0.11 |
| High school degree or below | 198 (66.4) | 121 (59.3) |  | 77 (32.8) | 69 (25.9) |  |
| University degree or above | 100 (33.6) | 83 (40.7) |  | 158 (67.2) | 197 (74.1) |  |
| Location, % |  |  | 0.90 |  |  | 0.97 |
| Rural | 95 (31.9) | 67 (32.8) |  | 87 (37.0) | 100 (37.6) |  |
| Urban | 203 (68.1) | 137 (67.2) |  | 148 (63.0) | 166 (62.4) |  |
| Smoking, % |  |  | 0.24 |  |  | 0.56 |
| No | 238 (79.9) | 153 (75.0) |  | 188 (80.0) | 206 (77.4) |  |
| Yes | 60 (20.1) | 51 (25.0) |  | 47 (20.0) | 60 (22.6) |  |
| Drinking, % |  |  | 0.61 |  |  | 0.23 |
| No | 278 (93.3) | 187 (91.7) |  | 225 (95.7) | 247 (92.9) |  |
| Yes | 20 (6.7) | 17 (8.3) |  | 10 (4.3) | 19 (7.1) |  |
| HBP, % |  |  | 0.68 |  |  | 1.00 |
| No | 278 (93.3) | 193 (94.6) |  | 233 (99.1) | 263 (98.9) |  |
| Yes | 20 (6.7) | 11 (5.4) |  | 2 (0.9) | 3 (1.1) |  |
| Diabetes, % |  |  | 1.00 |  |  | 0.55 |
| No | 284 (95.3) | 195 (95.6) |  | 233 (99.1) | 261 (98.1) |  |
| Yes | 14 (4.7) | 9 (4.4) |  | 2 (0.9) | 5 (1.9) |  |
| Obesity, % |  |  | 0.83 |  |  | 0.08 |
| No | 283 (95.0) | 192 (94.1) |  | 228 (97.0) | 248 (93.2) |  |
| Yes | 15 (5.0) | 12 (5.9) |  | 7 (3.0) | 18 (6.8) |  |
| Malignancy, % |  |  | 1.00 |  |  | 1.00 |
| No | 288 (96.6) | 198 (97.1) |  | 233 (99.1) | 263 (98.9) |  |
| Yes | 10 (3.4) | 6 (2.9) |  | 2 (0.9) | 3 (1.1) |  |
| CKD, % |  |  | 0.45 |  |  | 0.43 |
| No | 291 (97.7) | 196 (96.1) |  | 233 (99.1) | 266 (100) |  |
| Yes | 7 (2.3) | 8 (3.9) |  | 2 (0.9) | 0 (0) |  |
| Disease duration, % |  |  | 0.41 |  |  | 0.22 |
| <3years | 52 (17.4) | 35 (17.2) |  | 34 (14.5) | 32 (12.0) |  |
| 3-5years | 22 (7.4) | 18 (8.8) |  | 25 (10.6) | 40 (15.0) |  |
| 6-10years | 47 (15.8) | 22 (10.8) |  | 43 (18.3) | 38 (14.3) |  |
| 10-20years | 54 (18.1) | 47 (23.0) |  | 78 (33.2) | 104 (39.1) |  |
| 20 years+ | 123 (41.3) | 82 (40.2) |  | 55 (23.4) | 52 (19.5) |  |
| Drug therapy, % |  |  | 0.14 |  |  | **0.03** |
| No | 56 (18.8) | 28 (13.7) |  | 76 (32.3) | 63 (23.7) |  |
| Yes | 242 (81.2) | 176 (86.3) |  | 159 (67.7) | 203 (76.3) |  |
| Drug use duration, % |  |  | 0.63 |  |  | **0.01** |
| <6months | 42 (14.1) | 34 (16.7) |  | 35 (14.9) | 48 (18.0) |  |
| 6months-1year | 21 (7.0) | 16 (7.8) |  | 19 (8.1) | 21 (7.9) |  |
| 1-2years | 52 (17.4) | 40 (19.6) |  | 45 (19.1) | 32 (12.0) |  |
| 3-5years | 51 (17.1) | 42 (20.6) |  | 27 (11.5) | 48 (18.0) |  |
| 5-10years | 45 (15.1) | 26 (12.7) |  | 27 (11.5) | 38 (14.3) |  |
| >10years | 31 (10.4) | 18 (8.8) |  | 6 (2.6) | 16 (6.0) |  |
| No | 56 (18.8) | 28 (13.7) |  | 76 (32.3) | 63 (23.7) |  |
| GAD-7  [Median, IQR] | 2 (0,4) | 7 (5,10) | **<0.001** | 2 (0,4) | 7 (5,10) | **<0.001** |
| PHQ-9  [Median, IQR] | 2 (0,3) | 8 (6,11) | **<0.001** | 2 (0,3) | 8 (6,11) | **<0.001** |
| PSQI  [Median, IQR] | 5 (3,8) | 9 (7,12) | **<0.001** | 4 (3,6) | 7 (5,9) | **<0.001** |
| Anxiety, % |  |  | **<0.001** |  |  | - |
| No | 241 (80.9) | 45 (22.1) |  | 186 (79.1) | 41 (15.4) |  |
| Yes |  | 57 (19.1) |  | 49 (20.9) | 225 (84.6) |  |
| Sleep disorder, % |  |  | **<0.001** |  |  | **<0.001** |
| No | 156 (52.3) | 25 (12.3) |  | 173 (73.6) | 67 (25.2) |  |
| Yes | 142 (47.7) | 179 (87.7) |  | 62 (26.4) | 199 (74.8) |  |

Note: IQR: inter quartile range; HBP: high blood pressure; CKD: chronic kidney disease; GAD-7,7-tiem

Generalized Anxiety Disorder Scale; PHQ-9, Patient Health Questionnaire-9; PSQI, Pittsburgh sleep quality

index.
