# Supplementary material for: Mental health disorder in chronic liver disease: a questionnaire survey
Source: Front Psychiatry. 2024 Oct 25;15:1469372. doi: 10.3389/fpsyt.2024.1469372 (PMC11543405; doi:10.3389/fpsyt.2024.1469372)
Supplement: Supplementary file 6 [file Table6.docx]

Supplementary Table 6 Subgroup analysis of chronic liver disease and sleep disorder stratified by age.

| Variables | Age above median | | | Age below median | | |
| --- | --- | --- | --- | --- | --- | --- |
|  | Sleep disorder | | | Sleep disorder | | |
|  | No  (N=181) | Yes  (N=321) | *P* | No  (N=240) | Yes  (N=261) | *P* |
| Sex, % |  |  | **0.006** |  |  | 0.61 |
| Female | 44 (24.3) | 117 (36.4) |  | 83 (34.6) | 97 (37.2) |  |
| Male | 137 (75.7) | 204 (63.6) |  | 157 (65.4) | 164 (62.8) |  |
| BMI  [Median, IQR] | 23.0 (21.1,25.0) | 22.0 (19.8,24.0) | 0.96 | 23.2 (21.0,24.8) | 21.6 (19.9,24.2) | 0.95 |
| Education, % |  |  | 0.21 |  |  | 0.50 |
| High school degree or below | 122 (67.4) | 197 (61.4) |  | 66 (27.5) | 80 (30.7) |  |
| University degree or above | 59 (32.6) | 124 (38.6) |  | 174 (72.5) | 181 (69.3) |  |
| Location, % |  |  | 0.31 |  |  | 0.70 |
| Rural | 64 (35.4) | 98 (30.5) |  | 87 (36.3) | 100 (38.3) |  |
| Urban | 117 (64.6) | 223 (69.5) |  | 153 (63.8) | 161 (61.7) |  |
| Smoking, % |  |  | 0.32 |  |  | 0.41 |
| No | 136 (75.1) | 255 (79.4) |  | 193 (80.4) | 201 (77.0) |  |
| Yes | 45 (24.9) | 66 (20.6) |  | 47 (19.6) | 60 (23.0) |  |
| Drinking, % |  |  | 1.00 |  |  | 0.19 |
| No | 168 (92.8) | 297 (92.5) |  | 230 (95.8) | 242 (92.7) |  |
| Yes | 13 (7.2) | 24 (7.5) |  | 10 (4.2) | 19 (7.3) |  |
| HBP, % |  |  | 0.30 |  |  | 1.00 |
| No | 173 (95.6) | 298 (92.8) |  | 238 (99.2) | 258 (98.9) |  |
| Yes | 8 (4.4) | 23 (7.2) |  | 2 (0.8) | 3 (1.1) |  |
| Diabetes, % |  |  | 0.59 |  |  | 0.16 |
| No | 171 (94.5) | 308 (96.0) |  | 239 (99.6) | 255 (97.7) |  |
| Yes | 10 (5.5) | 13 (4.0) |  | 1 (0.4) | 6 (2.3) |  |
| Obesity, % |  |  | 0.61 |  |  | 0.54 |
| No | 173 (95.6) | 302 (94.1) |  | 230 (95.8) | 246 (94.3) |  |
| Yes | 8 (4.4) | 19 (5.9) |  | 10 (4.2) | 15 (5.7) |  |
| Malignancy, % |  |  | 1.00 |  |  | 0.42 |
| No | 175 (96.7) | 311 (96.9) |  | 239 (99.6) | 257 (98.5) |  |
| Yes | 6 (3.3) | 10 (3.1) |  | 1 (0.4) | 4 (1.5) |  |
| CKD, % |  |  | **0.03** |  |  | 0.44 |
| No | 180 (99.4) | 307 (95.6) |  | 238 (99.2) | 261 (100) |  |
| Yes | 1 (0.6) | 14 (4.4) |  | 2 (0.8) | 0 (0) |  |
| Disease duration, % |  |  | 0.18 |  |  | 0.25 |
| <3years | 24 (13.3) | 63 (19.6) |  | 32 (13.3) | 34 (13.0) |  |
| 3-5years | 15 (8.3) | 25 (7.8) |  | 30 (12.5) | 35 (13.4) |  |
| 6-10years | 32 (17.7) | 37 (11.5) |  | 47 (19.6) | 34 (13.0) |  |
| 10-20years | 34 (18.8) | 67 (20.9) |  | 78 (32.5) | 104 (39.8) |  |
| 20 years+ | 76 (42.0) | 129 (40.2) |  | 53 (22.1) | 54 (20.7) |  |
| Drug therapy, % |  |  | 0.57 |  |  | 0.50 |
| No | 28 (15.5) | 56 (17.4) |  | 70 (29.2) | 69 (26.4) |  |
| Yes | 153 (84.5) | 265 (82.6) |  | 170 (70.8) | 192 (73.6) |  |
| Drug use duration, % |  |  | 0.84 |  |  | 0.21 |
| <6months | 25 (13.8) | 51 (15.9) |  | 36 (15.0) | 47 (18.0) |  |
| 6months-1year | 16 (8.8) | 21 (6.5) |  | 19 (7.9) | 21 (8.0) |  |
| 1-2years | 32 (17.7) | 60 (18.7) |  | 46 (19.2) | 31 (11.9) |  |
| 3-5years | 33 (18.2) | 60 (18.7) |  | 33 (13.8) | 42 (16.1) |  |
| 5-10years | 30 (16.6) | 41 (12.8) |  | 29 (12.1) | 36 (13.8) |  |
| >10years | 17 (9.4) | 32 (10.0) |  | 7 (2.9) | 15 (5.7) |  |
| No | 28 (15.5) | 56 (17.4) |  | 70 (29.2) | 69 (26.4) |  |
| GAD-7  [Median, IQR] | 2 (0,4) | 5 (2,8) | **<0.001** | 3 (0,5) | 7 (4,9) | **<0.001** |
| PHQ-9  [Median, IQR] | 1 (0,3) | 2 (5,9) | **<0.001** | 2 (1,5) | 8 (5,10) | **<0.001** |
| PSQI  [Median, IQR] | 4 (3,4) | 9 (7,11) | **<0.001** | 3 (3,4) | 7 (4,10) | **<0.001** |
| Anxiety, % |  |  | **<0.001** |  |  | **<0.001** |
| No | 138 (76.2) | 148 (46.1) |  | 159 (66.3) | 68 (26.1) |  |
| Yes | 43 (23.8) | 173 (53.9) |  | 81 (33.8) | 193 (73.9) |  |
| Sleep disorder, % |  |  | **<0.001** |  |  | **<0.001** |
| No | 156 (86.2) | 142 (44.2) |  | 173 (72.1) | 62 (23.8) |  |
| Yes | 25 (13.8) | 179 (55.8) |  | 67 (27.9) | 199 (76.2) |  |

Note: IQR: inter quartile range; HBP: high blood pressure; CKD: chronic kidney disease; GAD-7,7-tiem

Generalized Anxiety Disorder Scale; PHQ-9, Patient Health Questionnaire-9; PSQI, Pittsburgh sleep quality

index.
