# Supplementary material for: Mental health disorder in chronic liver disease: a questionnaire survey
Source: Front Psychiatry. 2024 Oct 25;15:1469372. doi: 10.3389/fpsyt.2024.1469372 (PMC11543405; doi:10.3389/fpsyt.2024.1469372)
Supplement: Supplementary file 7 [file Table7.docx]

Supplementary Table 7 Subgroup analysis of chronic liver disease and anxiety stratified by education.

| Variables | High school degree or below | | | University degree or above | | |
| --- | --- | --- | --- | --- | --- | --- |
|  | Anxiety | | | Anxiety | | |
|  | No  (N=267) | Yes  (N=198) | *P* | No  (N=246) | Yes  (N=292) | *P* |
| Age  [Median, IQR] | 45 (37,50) | 42 (35,49) | **0.04** | 35 (30,44) | 34 (29,40.3) | 0.05 |
| BMI  [Median, IQR] | 22.3  (20.6, 24.2) | 22.6 (20.2,24.2) | 0.69 | 22.7 (20.8,24.9) | 22.4 (20.1,24.3) | **0.03** |
| Sex, % | 207 (72.4) | 134 (62.0) | **0.006** |  |  | 0.17 |
| Female | 81 (30.3) | 85 (42.9) |  | 72 (29.3) | 103 (35.3) |  |
| Male | 186 (69.7) | 113 (57.1) |  | 174 (70.7) | 189 (64.7) |  |
| Location, % |  |  | 0.74 |  |  | 0.31 |
| Rural | 139 (52.1) | 107 (54.0) |  | 42 (17.1) | 61 (20.9) |  |
| Urban | 128 (47.9) | 91 (46.0) |  | 204 (82.9) | 231 (79.1) |  |
| Smoking, % |  |  | 1.00 |  |  | 0.90 |
| No | 195 (73.0) | 144 (72.7) |  | 205 (83.3) | 241 (82.5) |  |
| Yes | 72 (27.0) | 54 (27.3) |  | 41 (16.7) | 51 (17.5) |  |
| Drinking, % |  |  | 0.44 |  |  | 0.15 |
| No | 243 (91.0) | 185 (93.4) |  | 237 (96.3) | 272 (93.2) |  |
| Yes | 24 (9.0) | 13 (6.6) |  | 9 (3.7) | 20 (6.8) |  |
| HBP, % |  |  | 0.63 |  |  | **0.03** |
| No | 251 (94.0) | 189 (95.5) |  | 237 (96.3) | 290 (99.3) |  |
| Yes | 16 (6.0) | 9 (4.5) |  | 9 (3.7) | 2 (0.7) |  |
| Diabetes, % |  |  | 0.78 |  |  | 0.77 |
| No | 255 (95.5) | 191 (96.5) |  | 240 (97.6) | 287 (98.3) |  |
| Yes | 12 (4.5) | 7 (3.5) |  | 6 (2.4) | 5 (1.7) |  |
| Obesity, % |  |  | 0.35 |  |  | 1.00 |
| No | 257 (96.3) | 186 (93.9) |  | 232 (94.3) | 276 (94.5) |  |
| Yes | 10 (3.7) | 12 (6.1) |  | 14 (5.7) | 16 (5.5) |  |
| Malignancy, % |  |  | 1.00 |  |  | 0.99 |
| No | 262 (98.1) | 194 (98.0) |  | 240 (97.6) | 286 (97.9) |  |
| Yes | 5 (1.9) | 4 (2.0) |  | 6 (2.4) | 6 (2.1) |  |
| CKD, % |  |  | 0.72 |  |  | 1.00 |
| No | 259 (97.0) | 190 (96.0) |  | 246 (100) | 291 (99.7) |  |
| Yes | 8 (3.0) | 8 (4.0) |  | 0 (0) | 1 (0.3) |  |
| Disease duration, % |  |  | 0.34 |  |  | **0.04** |
| <3years | 58 (21.7) | 45 (22.7) |  | 26 (10.6) | 24 (8.2) |  |
| 3-5years | 30 (11.2) | 23 (11.6) |  | 15 (6.1) | 37 (12.7) |  |
| 6-10years | 51 (19.1) | 24 (12.1) |  | 40 (16.3) | 35 (12.0) |  |
| 10-20years | 61 (22.8) | 54 (27.3) |  | 71 (28.9) | 97 (33.2) |  |
| 20 years+ | 67 (25.1) | 52 (26.3) |  | 94 (38.2) | 99 (33.9) |  |
| Drug therapy, % |  |  | 0.88 |  |  | 0.51 |
| No | 50 (18.7) | 36 (18.2) |  | 66 (26.8) | 71 (24.3) |  |
| Yes | 217 (81.3) | 162 (81.8) |  | 180 (73.2) | 221 (75.7) |  |
| Drug use duration, % |  |  | 0.41 |  |  | 0.80 |
| <6months | 42 (15.7) | 31 (15.7) |  | 39 (15.9) | 47 (16.1) |  |
| 6months-1year | 14 (5.2) | 17 (8.6) |  | 19 (7.7) | 27 (9.2) |  |
| 1-2years | 55 (20.6) | 37 (18.7) |  | 35 (14.2) | 42 (14.4) |  |
| 3-5years | 44 (16.5) | 42 (21.2) |  | 32 (13.0) | 50 (17.1) |  |
| 5-10years | 38 (14.2) | 25 (12.6) |  | 36 (14.6) | 37 (12.7) |  |
| >10years | 24 (9.0) | 10 (5.1) |  | 19 (7.7) | 18 (6.2) |  |
| No | 50 (18.7) | 36 (18.2) |  | 66 (26.8) | 71 (24.3) |  |
| GAD-7  [Median, IQR] | 1 (0,3) | 8 (6,11) | **<0.001** | 2 (0,3) | 7 (6,10) | **<0.001** |
| PHQ-9  [Median, IQR] | 1 (0,3) | 8 (5,12) | **<0.001** | 2 (0,4) | 8 (5,10) | **<0.001** |
| PSQI  [Median, IQR] | 5 (3,8) | 9 (6,11.8) | **<0.001** | 4.5 (3,7) | 7 (5,9.25) | **<0.001** |
| Depression, % |  |  | **<0.001** |  |  | **<0.001** |
| No | 226 (84.6) | 49 (24.7) |  | 201 (81.7) | 57 (19.5) |  |
| Yes | 41 (15.4) | 149 (75.3) |  | 45 (18.3) | 235 (80.5) |  |
| Sleep disorder, % |  |  | **<0.001** |  |  | **<0.001** |
| No | 144 (53.9) | 44 (22.2) |  | 153 (62.2) | 80 (27.4) |  |
| Yes | 123 (46.1) | 154 (77.8) |  | 93 (37.8) | 212 (72.6) |  |

Note: IQR: inter quartile range; HBP: high blood pressure; CKD: chronic kidney disease; GAD-7,7-tiem

Generalized Anxiety Disorder Scale; PHQ-9, Patient Health Questionnaire-9; PSQI, Pittsburgh sleep quality

index.
