# Supplementary material for: Mental health disorder in chronic liver disease: a questionnaire survey
Source: Front Psychiatry. 2024 Oct 25;15:1469372. doi: 10.3389/fpsyt.2024.1469372 (PMC11543405; doi:10.3389/fpsyt.2024.1469372)
Supplement: Supplementary file 8 [file Table8.docx]

Supplementary Table 8 Subgroup analysis of chronic liver disease and depression stratified by education.

| Variables | High school degree or below | | | University degree or above | | |
| --- | --- | --- | --- | --- | --- | --- |
|  | Depression | | | Depression | | |
|  | No  (N=275) | Yes  (N=190) | *P* | No  (N=258) | Yes  (N=280) | *P* |
| Age  [Median, IQR] | 45 (37,50) | 42 (35,48) | **0.02** | 36 (30,43.8) | 34 (29,40) | **0.01** |
| BMI  [Median, IQR] | 22.3  (20.6,24.1) | 22.8 (20.3,24.3) | 0.87 | 23 (20.8,24.9) | 22.2 (20.1,24.2) | **0.01** |
| Sex, % |  |  | 0.06 |  |  | 1.00 |
| Female | 88 (32.0) | 78 (41.1) |  | 84 (32.6) | 91 (32.5) |  |
| Male | 187 (68.0) | 112 (58.9) |  | 174 (67.4) | 189 (67.5) |  |
| Location, % |  |  | 0.13 |  |  | 0.39 |
| Rural | 137 (49.8) | 109 (57.4) |  | 45 (17.4) | 58 (20.7) |  |
| Urban | 138 (50.2) | 81 (42.6) |  | 213 (82.6) | 222 (79.3) |  |
| Smoking, % |  |  | **0.01** |  |  | 1.00 |
| No | 212 (77.1) | 127 (66.8) |  | 214 (82.9) | 232 (82.9) |  |
| Yes | 63 (22.9) | 63 (33.2) |  | 44 (17.1) | 48 (17.1) |  |
| Drinking, % |  |  | 0.89 |  |  | 0.09 |
| No | 254 (92.4) | 174 (91.6) |  | 249 (96.5) | 260 (92.9) |  |
| Yes | 21 (7.6) | 16 (8.4) |  | 9 (3.5) | 20 (7.1) |  |
| HBP, % |  |  | 0.91 |  |  | 0.18 |
| No | 261 (94.9) | 179 (94.2) |  | 250 (96.9) | 277 (98.9) |  |
| Yes | 14 (5.1) | 11 (5.8) |  | 8 (3.1) | 3 (1.1) |  |
| Diabetes, % |  |  | 0.73 |  |  | 0.89 |
| No | 265 (96.4) | 181 (95.3) |  | 252 (97.7) | 275 (98.2) |  |
| Yes | 10 (3.6) | 9 (4.7) |  | 6 (2.3) | 5 (1.8) |  |
| Obesity, % |  |  | 0.12 |  |  | 0.74 |
| No | 266 (96.7) | 177 (93.2) |  | 245 (95.0) | 263 (93.9) |  |
| Yes | 9 (3.3) | 13 (6.8) |  | 13 (5.0) | 17 (6.1 |  |
| Malignancy, % |  |  | 0.42 |  |  | 0.88 |
| No | 268 (97.5) | 188 (98.9) |  | 253 (98.1) | 273 (97.5) |  |
| Yes | 7 (2.5) | 2 (1.1) |  | 5 (1.9) | 7 (2.5) |  |
| CKD, % |  |  | 1.00 |  |  | 1.00 |
| No | 266 (96.7) | 183 (96.3) |  | 258 (100) | 279 (99.6) |  |
| Yes | 9 (3.3) | 7 (3.7) |  | 0 (0) | 1 (0.4) |  |
| Disease duration, % |  |  | 0.94 |  |  | **0.002** |
| <3years | 60 (21.8) | 43 (22.6) |  | 26 (10.1) | 24 (8.6) |  |
| 3-5years | 30 (10.9) | 23 (12.1) |  | 17 (6.6) | 35 (12.5) |  |
| 6-10years | 45 (16.4) | 30 (15.8) |  | 45 (17.4) | 30 (10.7) |  |
| 10-20years | 66 (24.0) | 49 (25.8) |  | 66 (25.6) | 102 (36.4) |  |
| 20 years+ | 74 (26.9) | 45 (23.7) |  | 104 (40.3) | 89 (31.8) |  |
| Drug therapy, % |  |  | **0.05** |  |  | 0.15 |
| No | 59 (21.5) | 27 (14.2) |  | 73 (28.3) | 64 (22.9) |  |
| Yes | 216 (78.5) | 163 (85.8) |  | 185 (71.7) | 216 (77.1) |  |
| Drug use duration, % |  |  | 0.20 |  |  | 0.37 |
| <6months | 39 (14.2) | 34 (17.9) |  | 38 (14.7) | 48 (17.1) |  |
| 6months-1year | 15 (5.5) | 16 (8.4) |  | 25 (9.7) | 21 (7.5) |  |
| 1-2years | 59 (21.5) | 33 (17.4) |  | 38 (14.7) | 39 (13.9) |  |
| 3-5years | 46 (16.7) | 40 (21.1) |  | 32 (12.4) | 50 (17.9) |  |
| 5-10years | 35 (12.7) | 28 (14.7) |  | 37 (14.3) | 36 (12.9) |  |
| >10years | 22 (8.0) | 12 (6.3) |  | 15 (5.8) | 22 (7.9) |  |
| No | 59 (21.5) | 27 (14.2) |  | 73 (28.3) | 64 (22.9) |  |
| GAD-7  [Median, IQR] | 1 (0,3) | 8 (5,11) | **<0.001** | 2 (0,4) | 7 (5,10) | **<0.001** |
| PHQ-9  [Median, IQR] | 1 (0,3) | 8 (6.25,12) | **<0.001** | 2 (0,3) | 8 (6,10) | **<0.001** |
| PSQI  [Median, IQR] | 5 (3,7) | 9 (7,12) | **<0.001** | 4 (3,6) | 7 (6,10) | **<0.001** |
| Anxiety, % |  |  | **<0.001** |  |  | **<0.001** |
| No | 226 (82.2) | 41 (21.6) |  | 201 (77.9) | 45 (16.1) |  |
| Yes | 49 (17.8) | 149 (78.4) |  | 57 (22.1) | 235 (83.9) |  |
| Sleep disorder, % |  |  | **<0.001** |  |  | **<0.001** |
| No | 160 (58.2) | 28 (14.7) |  | 169 (65.5) | 64 (22.9) |  |
| Yes | 115 (41.8) | 162 (85.3) |  | 89 (34.5) | 216 (77.1) |  |

Note: IQR: inter quartile range; HBP: high blood pressure; CKD: chronic kidney disease; GAD-7,7-tiem

Generalized Anxiety Disorder Scale; PHQ-9, Patient Health Questionnaire-9; PSQI, Pittsburgh sleep quality

index.
