# Supplementary material for: Mental health disorder in chronic liver disease: a questionnaire survey
Source: Front Psychiatry. 2024 Oct 25;15:1469372. doi: 10.3389/fpsyt.2024.1469372 (PMC11543405; doi:10.3389/fpsyt.2024.1469372)
Supplement: Supplementary file 9 [file Table9.docx]

Supplementary Table 9 Subgroup analysis of chronic liver disease and depression sleep disorder by education.

| Variables | High school degree or below | | | University degree or above | | |
| --- | --- | --- | --- | --- | --- | --- |
|  | Sleep disorder | | | Sleep disorder | | |
|  | No  (N=188) | Yes  (N=277) | *P* | No  (N=233) | Yes  (N=305) | *P* |
| Age  [Median, IQR] | 42 (33,48) | 44 (37,50) | **0.02** | 33 (29,39) | 36 (30,45) | **<0.001** |
| BMI  [Median, IQR] | 22.3 (20.3,24.1) | 22.6 (20.5,24.2) | 0.66 | 22.3 (20.4,24.6) | 22.7 (20.7,24.7) | 0.65 |
| Sex, % |  |  | **0.04** |  |  | 0.43 |
| Female | 56 (29.8) | 110 (39.7) |  | 71 (30.5) | 104 (34.1) |  |
| Male | 132 (70.2) | 167 (60.3) |  | 162 (69.5) | 201 (65.9) |  |
| Location, % |  |  | 0.44 |  |  | 0.68 |
| Rural | 104 (55.3) | 142 (51.3) |  | 47 (20.2) | 56 (18.4) |  |
| Urban | 84 (44.7) | 135 (48.7) |  | 186 (79.8) | 249 (81.6) |  |
| Smoking, % |  |  | 0.74 |  |  | 0.94 |
| No | 135 (71.8) | 204 (73.6) |  | 194 (83.3) | 252 (82.6) |  |
| Yes | 53 (28.2) | 73 (26.4) |  | 39 (16.7) | 53 (17.4) |  |
| Drinking, % |  |  | 0.87 |  |  | 0.24 |
| No | 174 (92.6) | 254 (91.7) |  | 224 (96.1) | 285 (93.4) |  |
| Yes | 14 (7.4) | 23 (8.3) |  | 9 (3.9) | 20 (6.6) |  |
| HBP, % |  |  | 0.13 |  |  | 0.87 |
| No | 182 (96.8) | 258 (93.1) |  | 229 (98.3) | 298 (97.7) |  |
| Yes | 6 (3.2) | 19 (6.9) |  | 4 (1.7) | 7 (2.3) |  |
| Diabetes, % |  |  | 1.00 |  |  | 0.44 |
| No | 180 (95.7) | 266 (96.0) |  | 230 (98.7) | 297 (97.4) |  |
| Yes | 8 (4.3) | 11 (4.0) |  | 3 (1.3) | 8 (2.6) |  |
| Obesity, % |  |  | 0.54 |  |  | 0.57 |
| No | 181 (96.3) | 262 (94.6) |  | 222 (95.3) | 286 (93.8) |  |
| Yes | 7 (3.7) | 15 (5.4) |  | 11 (4.7) | 19 (6.2) |  |
| Malignancy, % |  |  | 1.00 |  |  | 0.32 |
| No | 184 (97.9) | 272 (98.2) |  | 230 (98.7) | 296 (97.0) |  |
| Yes | 4 (2.1) | 5 (1.8) |  | 3 (1.3) | 9 (3.0) |  |
| CKD, % |  |  | 0.12 |  |  | 1.00 |
| No | 185 (98.4) | 264 (95.3) |  | 233 (100) | 304 (99.7) |  |
| Yes | 3 (1.6) | 13 (4.7) |  | 0 (0) | 1 (0.3) |  |
| Disease duration, % |  |  | 0.20 |  |  | 0.17 |
| <3years | 36 (19.1) | 67 (24.2) |  | 20 (8.6) | 30 (9.8) |  |
| 3-5years | 26 (13.8) | 27 (9.7) |  | 19 (8.2) | 33 (10.8) |  |
| 6-10years | 37 (19.7) | 38 (13.7) |  | 42 (18.0) | 33 (10.8) |  |
| 10-20years | 43 (22.9) | 72 (26.0) |  | 69 (29.6) | 99 (32.5) |  |
| 20 years+ | 46 (24.5) | 73 (26.4) |  | 83 (35.6) | 110 (36.1) |  |
| Drug therapy, % |  |  | 0.59 |  |  | 0.74 |
| No | 37 (19.7) | 49 (17.7) |  | 61 (26.2) | 76 (24.9) |  |
| Yes | 151 (80.3) | 228 (82.3) |  | 172 (73.8) | 229 (75.1) |  |
| Drug use duration, % |  |  | 0.42 |  |  | 0.93 |
| <6months | 25 (13.3) | 48 (17.3) |  | 36 (15.5) | 50 (16.4) |  |
| 6months-1year | 12 (6.4) | 19 (6.9) |  | 23 (9.9) | 23 (7.5) |  |
| 1-2years | 45 (23.9) | 47 (17.0) |  | 33 (14.2) | 44 (14.4) |  |
| 3-5years | 33 (17.6) | 53 (19.1) |  | 33 (14.2) | 49 (16.1) |  |
| 5-10years | 26 (13.8) | 37 (13.4) |  | 33 (14.2) | 40 (13.1) |  |
| >10years | 10 (5.3) | 24 (8.7) |  | 14 (6.0) | 23 (7.5) |  |
| No | 37 (19.7) | 49 (17.7) |  | 61 (26.2) | 76 (24.9) |  |
| GAD-7  [Median, IQR] | 1.5 (0,4) | 5 (2,9) | **<0.001** | 3 (1,5) | 6 (4,9) | **<0.001** |
| PHQ-9  [Median, IQR] | 1 (0,3) | 6 (2,10) | **<0.001** | 2 (1,5) | 7 (4,9) | **<0.001** |
| PSQI  [Median, IQR] | 3 (3,4) | 9 (7,11) | **<0.001** | 3 (3,5) | 8 (7,10) | **<0.001** |
| Anxiety, % |  |  | **<0.001** |  |  | **<0.001** |
| No | 144 (76.6) | 123 (44.4) |  | 153 (65.7) | 93 (30.5) |  |
| Yes | 44 (23.4) | 154 (55.6) |  | 80 (34.3) | 212 (69.5) |  |
| Depression, % |  |  | **<0.001** |  |  | **<0.001** |
| No | 160 (85.1) | 115 (41.5) |  | 169 (72.5) | 89 (29.2) |  |
| Yes | 28 (14.9) | 162 (58.5) |  | 64 (27.5) | 216 (70.8) |  |

Note: IQR: inter quartile range; HBP: high blood pressure; CKD: chronic kidney disease; GAD-7,7-tiem

Generalized Anxiety Disorder Scale; PHQ-9, Patient Health Questionnaire-9; PSQI, Pittsburgh sleep quality

index.
