# Supplementary material for: Mental health disorder in chronic liver disease: a questionnaire survey
Source: Front Psychiatry. 2024 Oct 25;15:1469372. doi: 10.3389/fpsyt.2024.1469372 (PMC11543405; doi:10.3389/fpsyt.2024.1469372)
Supplement: Supplementary file 10 [file Table10.docx]

Table S1 Univariate logistic regression analysis of factors that affect the anxiety of patients with chronic liver disease.

| Characteristics | Beta | S.E. | z-value | Pr(>\|z\|) |
| --- | --- | --- | --- | --- |
| Sex (male) | -0.38 | 0.13 | -2.85 | **0.004** |
| Age | -0.024 | 0.0063 | -3.78 | **<0.001** |
| BMI | -0.04240 | 0.02196 | -1.931 | 0.054 |
| Education  (University degree or above) | 0.47 | 0.13 | 3.69 | **<0.001** |
| Location (Urban) | 0.044 | 0.13 | 0.33 | 0.74 |
| Smoking | -0.035 | 0.15 | -0.23 | 0.82 |
| Drinking | 0.049 | 0.25 | 0.19 | 0.85 |
| HBP | -0.80 | 0.37 | -2.18 | **0.03** |
| Diabetes | -0.37 | 0.38 | -0.98 | 0.33 |
| Obesity | 0.21 | 0.29 | 0.74 | 0.46 |
| Malignancy | -0.051 | 0.44 | -0.11 | 0.91 |
| CKD | 0.17 | 0.49 | 0.34 | 0.73 |
| Disease duration | -0.20 | 0.16 | -1.21 | 0.23 |
| Drug therapy | 0.080 | 0.15 | 0.53 | 0.60 |
| Drug use duration | -0.038 | 0.16 | -0.24 | 0.81 |

Abbreviations: CI, confidence internal; S.E., standard error; BMI, body mass index; HBP, high blood pressure; CKD, chronic kidney disease.

Table S2 Multivariate logistic regression analysis of factors that affect the anxiety of patients with chronic liver disease.

| Characteristics | Beta | S.E. | z-value | Pr(>\|z\|) | OR (95%CI) |
| --- | --- | --- | --- | --- | --- |
| Sex (male) | -0.39 | 0.14 | -2.88 | **0.004** | 0.68(0.52-0.88) |
| Age | -0.016 | 0.0068 | -2.30 | **0.02** | 0.98(0.97-0.99) |
| Education  (University degree or above) | 0.37 | 0.14 | 2.71 | **0.007** | 1.45(1.11-1.89) |
| HBP | -0.54 | 0.38 | -1.44 | 0.15 | - |

Abbreviations: CI, confidence internal; S.E., standard error; HBP, high blood pressure.

Table S3 Univariate logistic regression analysis of factors that affect the depression of patients with chronic liver disease.

| Characteristics | Beta | S.E. | z-value | Pr(>\|z\|) |
| --- | --- | --- | --- | --- |
| Sex (male) | -0.16 | 0.13 | -1.23 | 0.22 |
| Age | -0.027 | 0.0064 | -4.29 | **<0.001** |
| BMI | -0.037 | 0.021 | -1.680 | 0.093 |
| Education  (University degree or above) | 0.45 | 0.13 | 3.53 | **<0.001** |
| Location (Urban) | -0.061 | 0.13 | -0.460 | 0.65 |
| Smoking | 0.21 | 0.15 | 1.36 | 0.18 |
| Drinking | 0.33 | 0.26 | 1.29 | 0.20 |
| HBP | -0.34 | 0.35 | -0.97 | 0.33 |
| Diabetes | -0.008 | 0.37 | -0.021 | 0.98 |
| Obesity | 0.46 | 0.29 | 1.60 | 0.11 |
| Malignancy | -0.16 | 0.45 | -0.37 | 0.71 |
| CKD | 0.008 | 0.49 | 0.02 | 0.99 |
| Disease duration | -0.25 | 0.16 | -1.53 | 0.13 |
| Drug therapy | 0.16 | 0.15 | 1.07 | 0.28 |
| Drug use duration | 0.46 | 0.26 | 1.8 | 0.07 |

Abbreviations: CI, confidence internal; S.E., standard error; BMI, body mass index; HBP, high blood pressure; CKD, chronic kidney disease.

Table S4 Multivariate logistic regression analysis of factors that affect the depression of patients with chronic liver disease.

| Characteristics | Beta | S.E. | z-value | Pr(>\|z\|) | OR (95%CI) |
| --- | --- | --- | --- | --- | --- |
| Age | -0.022 | 0.0067 | -3.36 | **<0.001** | 0.98 (0.96-0.99) |
| Education  (University degree or above) | 0.30 | 0.14 | 2.25 | **0.02** | 1.36 (1.04-1.77) |

Abbreviations: CI, confidence internal; S.E., standard error.

Table S5 Univariate logistic regression analysis of factors that affect the sleep disorder of patients with chronic liver disease.

| Characteristics | Beta | S.E. | z-value | Pr(>\|z\|) |
| --- | --- | --- | --- | --- |
| Sex (male) | -0.30 | 0.14 | -2.18 | **0.03** |
| Age | 0.031 | 0.007 | 4.71 | **<0.001** |
| BMI | 0.008 | 0.022 | 0.394 | 0.69 |
| Education  (University degree or above) | -0.12 | 0.12845 | -0.92 | 0.36 |
| Location (Urban) | 0.081 | 0.13 | 0.61 | 0.54 |
| Smoking | -0.012 | 0.16 | -0.077 | 0.94 |
| Drinking | 0.32 | 0.27 | 1.21 | 0.23 |
| HBP | 0.65 | 0.38 | 1.73 | 0.08 |
| Diabetes | 0.23 | 0.38 | 0.60 | 0.55 |
| Obesity | 0.33 | 0.30 | 1.10 | 0.27 |
| Malignancy | 0.38 | 0.47 | 0.81 | 0.42 |
| CKD | 1.23 | 0.64 | 1.93 | 0.05 |
| Disease duration | -0.59 | 0.33 | -1.80 | 0.07 |
| Drug therapy | 0.14 | 0.15 | 0.90 | 0.37 |
| Drug use duration | -0.21 | 0.21 | -1.02 | 0.31 |

Abbreviations: CI, confidence internal; S.E., standard error; BMI, body mass index; HBP, high blood pressure; CKD, chronic kidney disease.

Table S6 Multivariate logistic regression analysis of factors that affect the sleep disorder of patients with chronic liver disease.

| Characteristics | Beta | S.E. | z-value | Pr(>\|z\|) | OR (95%CI) |
| --- | --- | --- | --- | --- | --- |
| Age | 0.03 | 0.007 | 4.80 | **<0.001** | 1.03 (1.01-1.05) |
| Sex (male) | -0.33 | 0.14 | -2.38 | **0.02** | 0.72 (0.55-0.94) |

Abbreviations: CI, confidence internal; S.E., standard error.
